# Automatic measurement of air gap for proton therapy using orthogonal x‐ray imaging with radiopaque wires

**DOI:** 10.1002/acm2.12509

**Published:** 2018-12-16

**Authors:** Pavitra Ramesh, Wei Song, Hongbin Cao, Yanqun Zhao, Rahul Parikh, Joseph Weiner, Xiao Wang, Ke Nie, Ning Yue, Yin Zhang

**Affiliations:** ^1^ Department of Radiation Oncology Rutgers‐Cancer Institute of New Jersey Rutgers‐Robert Wood Johnson University Hospital Rutgers‐The State University of New Jersey New Brunswick NJ USA; ^2^ Department of Radiation Oncology Jiangsu Province Hospital of TCM Nanjing Jiangsu China; ^3^ Department of Radiation Oncology School of Medicine Renji Hospital Shanghai Jiao Tong University Shanghai China; ^4^ Sichuan Cancer Center School of Medicine Sichuan Cancer Hospital & Institute University of Electronic Science and Technology of China Chengdu China

**Keywords:** air gap, orthogonal kV images, proton therapy

## Abstract

**Purpose:**

The main objective of this study was to develop a technique to accurately determine the air gap between the end of the proton beam compensator and the body of the patient in proton radiotherapy.

**Methods:**

Orthogonal x‐ray image‐based automatic coordinate reconstruction was used to determine the air gap between the patient body surface contour and the end of beam nozzle in proton radiotherapy. To be able to clearly identify the patient body surface contour on the orthogonal images, a radiopaque wire was placed on the skin surface of the patient as a surrogate. In order to validate this method, a Rando^®^ head phantom was scanned and five proton plans were generated on a Mevion S250 Proton machine with various air gaps in Varian Eclipse Treatment Planning Systems (TPS). When setting up the phantom in a treatment room, a solder wire was placed on the surface of the phantom closest to the beam nozzle with the knowledge of the beam geometry in the plan. After the phantom positioning was verified using orthogonal kV imaging, the last pair of setup kV images was used to segment the solder wire and the in‐room coordinates of the wire were reconstructed using a back‐projection algorithm. Using the wire as a surrogate of the body surface, we calculated the air gaps by finding the minimum distance between the reconstructed wire and the end of the compensator. The methodology was also verified and validated on clinical cases.

**Results:**

On the phantom study, the air gap values derived with the automatic reconstruction method were found to be within 1.1 mm difference from the planned values for proton beams with air gaps of 85.0, 100.0, 150.0, 180.0, and 200.0 mm. The reconstruction technique determined air gaps for a patient in two clinical treatment sessions were 38.4 and 41.8 mm, respectively, for a 40 mm planned air gap, and confirmed by manual measurements. There was strong agreement between the calculated values and the automatically measured values, and between the automatically and manually measured values.

**Conclusions:**

An image‐based automatic method has been developed to conveniently determine the air gap of a proton beam, directly using the orthogonal images for patient positioning without adding additional imaging dose to the patient. The method provides an objective, accurate, and efficient way to confirm the target depth at treatment to ensure desired target coverage and normal tissue sparing.

## 1 INTRODUCTION

Delivering correct amount of dosage to the tumor target without affecting surrounding healthy tissue is an important mission in radiation oncology.^1^ The advantage of using proton therapy over photon therapy is that proton beams can deposit high dosage of radiation exclusively over a region at a certain depth below the skin surface and the normal tissue beyond the distal end of the beam receives minimal dose.^2^, ^3^, ^4^ This physical characteristic of proton beams requires high precision in determining the target depth.^5^ Any substantial changes of the target depth, such as the external body contour changes due to weight loss, may lead to potential target miss or significant damage to the normal tissues which would otherwise be spared.^6^, ^7^ To ensure accurate setup during each fractioned treatment and target depth, the air gap from the patient's skin surface must be measured and checked after patient positioning and prior to the beam delivery to avoid potential mistreatment.

With the exception of limited number of proton centers equipped with three‐dimensional (3D) imaging positioning systems,^8^ the two‐dimensional (2D) orthogonal imaging‐based system is still widely employed at many proton centers for the purpose of patient positioning. Unlike the 3D imaging system which can directly provide target depth information, with the 2D imaging system, the target depth information can only be indirectly derived by measuring the air gap between patient surface and beam nozzle prior to treatment. Currently, measurement of the air gap is mostly manually conducted using a ruler, and the measurement can be subjective and inefficient. It would be desirable to utilize the same imaging process for patient treatment position purpose to determine the air gap, at no expense of additional imaging time and dose, even for the proton machines equipped with only two‐dimensional kilo‐voltage (kV) imaging systems. The goal of this study is to develop an orthogonal imaging‐based automatic method for the determination of the air gap at proton therapy treatment.

## 2 MATERIALS AND METHODS

**Figure 1:**
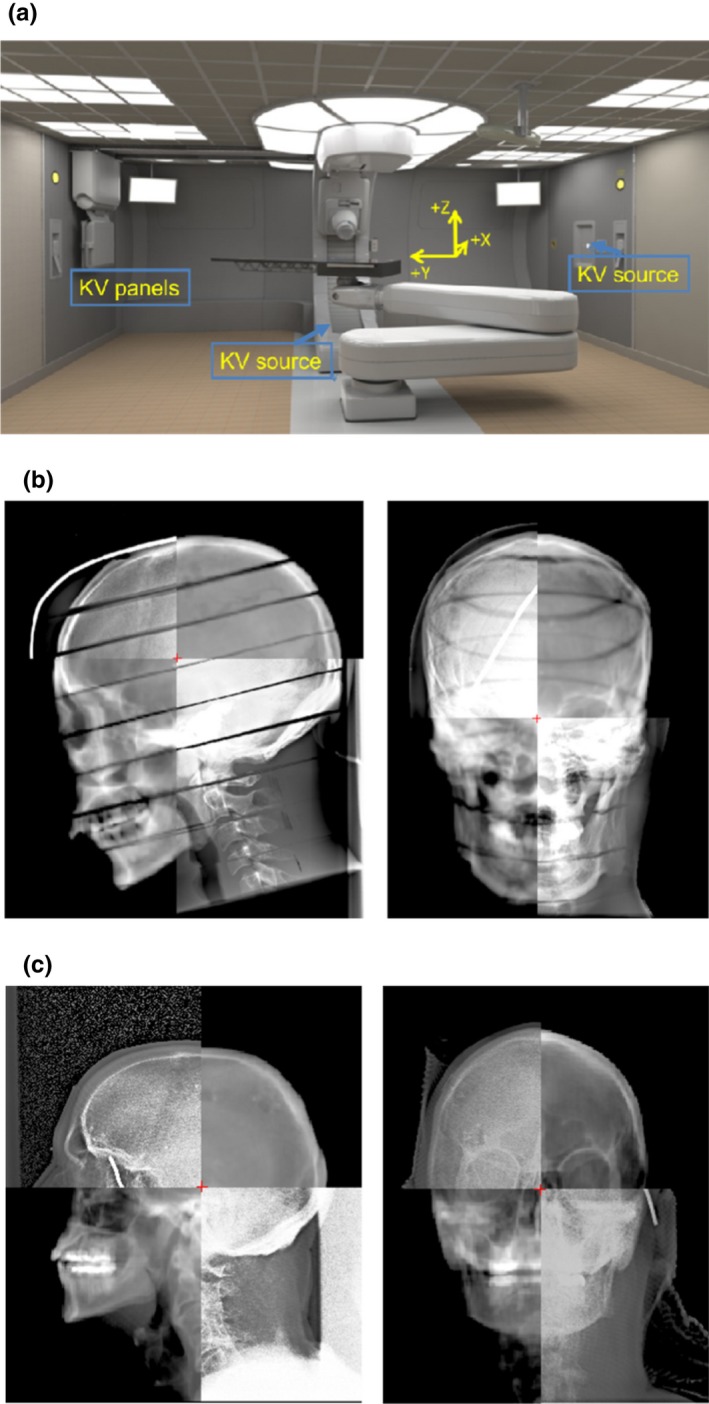
(a) Room setup of the Mevion S250™ proton therapy machine. (b) Left lateral (left) and post‐anterior (right) kV images of the phantom head blended with reference kV images. (c) Left lateral (left) and PA (right) kV images of a proton patient with brain leision, blended with reference kV images.

The method is based on the idea that coordinates of a linear object imaged on the orthogonal images can be reconstructed.^9^ Since the air gap is measured from the patient skin surface and it is normally difficult to clearly identify soft tissue on x‐ray images, a radiopaque wire can be placed along the patient skin surface during the patient treatment setup in the general location where the proton beam is aimed at (this wire will be removed prior to beam delivery). The developed method was first validated using a Rando^®^ phantom on a Mevion S250 proton therapy machine, as shown in Fig. 1(a), and subsequently validated in two consecutive treatment sessions for a proton patient.

The phantom was scanned on a CT simulator (GE Lightspeed^TM^ 16) for the simulation and planning. Multiple proton treatment plans with various air gaps were created based on the CT images for the purpose of method development and validation. The plans were generated with Eclipse Treatment Planning System (Version 11, Varian Medical Systems, Pal Alto, CA).

Based on the treatment plans, the phantom was positioned on the proton machine treatment couch. A radiopaque wire was placed on the phantom surface where a proton beam was aimed at and pre‐identified on the planning CT images. We obtained two kV images from the post‐anterior (PA) angle and left lateral (LLAT) angle projections using the imaging system equipped on the proton machine to position the phantom to the planned position. The pair of the acquired orthogonal images is shown in Fig. 1(b). The images were acquired and saved in Digital Imaging and Communication in Medicine (DICOM) format. A region growing algorithm that relied on the image intensity and its associated gradient was used to automatically segment the wire from the orthogonal image. Then, the coordinates of the wire from 2D orthogonal images were reconstructed into a 3D space.

**Figure 2:**
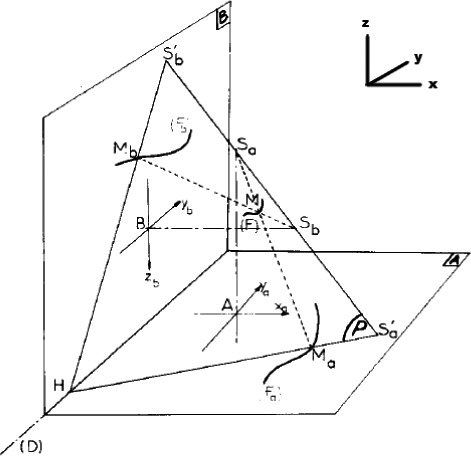
Setup and matching of corresponding points for two orthogonal films and relative locations of the kV panels and sources.^9^ *Note*. The actual coordinate system used in DICOM needs to be rotated to match the coordinate system shown above.

An in‐house built fully automatic program was developed in MATLAB to reconstruct, plot, and analyze the data. In the image digital processing, the LLAT and PA images were represented as large binary matrices by the processing algorithm. The cells of these matrices showed a value of 1 for every piece of the wire identified on the LLAT and PA images and a value of 0 for areas of the images that did not contain the wire. The centerlines of the wire projection on the LLAT and PA films were identified and fitted using smoothing spline model type. To reconstruct the wire placed on the patient body surface, the wire projection on the PA film was first divided into a series of points. The reconstruction method for one of these points, M_a_ (x_a_, y_a_) on the PA image is described as follow. To reconstruct the point M (Fig. 2) that was imaged as M_a_ on the PA film, we have to find its homologous point on the lateral film, M_b_ (y_b_, z_b_), assuming this lateral point rests on the intersection of the wire and the plane defined by: the lateral source position, the PA source position, and the point M_a_. Both film coordinates must satisfy the Eq. (1), with d_1_, d_2_, d_3_, and d_4_ as constants for the PA x‐ray source to isocenter distance (158.4 cm), the isocenter to PA imaging panel distance (48.3 cm), the lateral x‐ray source to isocenter distance (303.22 cm), and the isocenter to lateral imaging panel distance (49.5 cm), respectively:

(1) $$\left[\left(d_{1}+d_{2}\right)d_{3}-d_{1}x_{a}\right]y_{b}+d_{3}y_{a}z_{b}=\left(d_{3}+d_{4}\right)d_{1}y_{a}$$

An illustration of the setup of the x‐ray sources and images, as well as the projection of the wire is shown in Fig. 2. The measurements for the orthogonal reconstruction of the wire that are needed for the calculation are also shown in the diagram.

Once we know the coordinates of the chosen point on both two‐dimensional films, M_a_ (x_a_, y_a_) and M_b_ (y_b_, z_b_), we use the reconstruction algorithm to find the coordinates of the point M (x_m_, y_m_, z_m_) on the wire relative to the isocenter in three‐dimensional space. These coordinates can be found by finding the intersection of two lines, one from M_a_ to the PA source point (S_a_) and the other from M_b_ to the lateral source (S_b_). The general algorithm becomes:

(2) $$\begin{array}{cc} C & =\frac{x_{a}y_{b}}{y_{a}};\\ x_{m} & =-d_{4}+\frac{\left(d_{3}+d_{4}\right)\left(d_{4}+c\right)}{d_{3}+d_{4}+c};\\ y_{m} & =y_{b}-\frac{\left(d_{4}+c\right)y_{b}}{d_{3}+d_{4}+c};\\ z_{m} & =z_{b}-\frac{\left(d_{4}+c\right)z_{b}}{d_{3}+d_{4}+c}. \end{array}$$

Exceptions to this algorithm are needed, for example, when y_a_, y_b_, or both are equal to zero. These exceptions are added into the program separately. We follow this algorithm for all points identified for the centerlines of wire projected on both films until we have reconstructed the centerline of the wire in three‐dimensional space. We align the points on the wire and find the shortest distance from the wire to the end of the snout or compensator tray. The code searches through the newly reconstructed points on the wire, calculates the distance from each of these points to the compensator tray, and returns the smallest value.

Using the above discussed algorithm, our computer program takes the two‐dimensional LLAT and PA positioning images, as well as the planning RT Structure set and treatment RT plan as inputs. The outputs of the computer program consist of the coordinates of the points on the wire, the calculated air gap using the points on the wire, the planned air gap using the corresponding DICOM body contour sequence, and a three‐dimensional plot of the body contour, the wire placed on top of it, the isocenter, and the compensator tray or snout.

## 3 RESULTS

**Figure 3:**
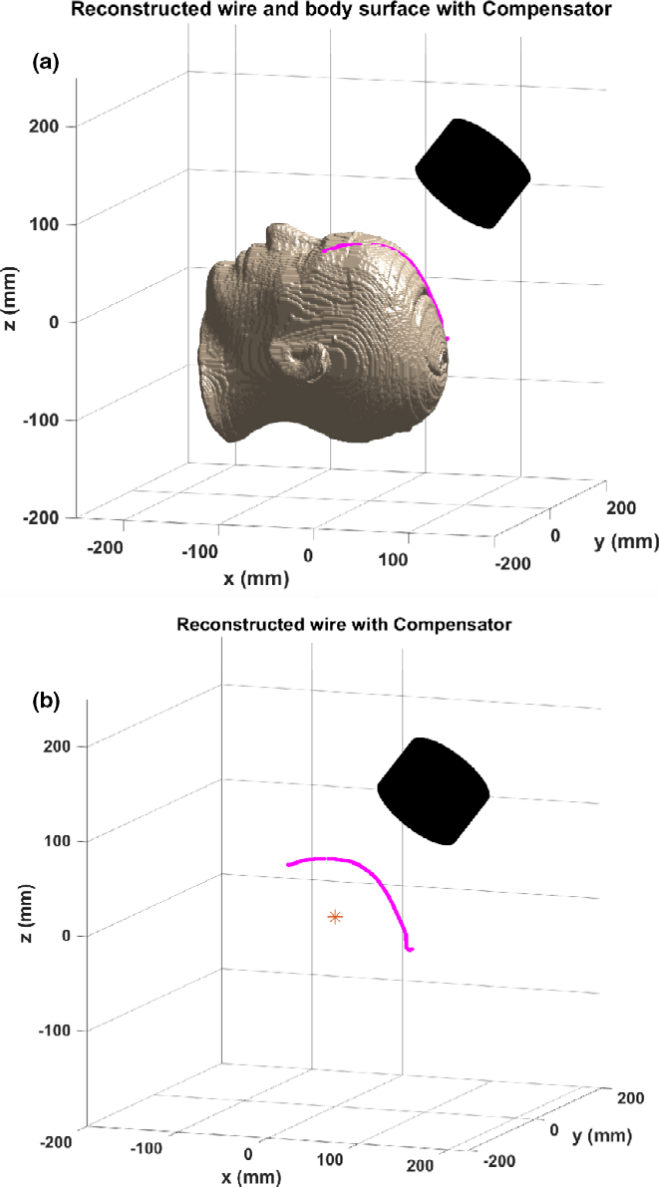
(a) Wire placement and surface contour of the Rando^®^ head along with the end of the compensator. (b) Reconstruction of the wire relative to the isocenter.

Figure 3(a) shows the outputs from the program. The surface of the phantom head is created with the body contour data from the planning system. The reconstructed wire and the compensator are represented by the magenta line and the black cylinder, respectively, relative to the red isocenter. An image for the reconstructed wire without the surface contour is given in Fig. 3(b). The derived air gap results are shown in Table 1. Listed are the air gap values computed from the treatment plans, the derived corresponding results using the wire reconstruction method, and the calculated air gap values accounting for the thickness of the wire. Taking the wire thickness into account, good agreements were found among the true values of air gaps and the results derived with the developed algorithm and software program. To validate the methodology in clinical treatment, it was applied to two clinical treatment sessions and the measured air gaps were 38.4 and 41.8 mm, respectively, compared to a 40 mm planned air gap, after taking the wire thickness into account.

**Table 1:** Measurements of the air gap

Planned values (mm)	Program outputs (mm)	Calculated air gap (mm)
85	82.62	83.97
100	97.62	98.97
150	147.62	148.97
180	177.62	178.97
200	197.62	198.97

## 4 DISCUSSION

In this study, we have developed an orthogonal kV imaging‐based method to automatically reconstruct a wire from the digital images and to derive the proton beam air gaps at treatment. The method can be used to automatically, although indirectly, confirm and verify treatment depth. Excellent agreements were found for the results. With the more accurately and objective measurement method as developed in this study, it is possible to monitor the variation of the air gap during treatment session and decide on the appropriate tolerance of the air gap for different sites. The planned distances, program outputs, and corresponding calculated air gaps are presented in Table 1. The differences between the derived air gap values and the expected values were within 1.1 mm. The excellent agreements demonstrate that the developed algorithm and the software program are precise and are able to handle treatments of various air gap values. The derived air gap values are objective unlike those values manually measured with a ruler. Once the patient is positioned using the orthogonal kV images, the air gap between the patient surface relative to the snout is verified.

The accuracy of identification and distinguishing of the wire from the images can be compromised by the digital noises in the DICOM files. The current approach to resolve the uncertainty is visual confirmation on the images after the reconstruction. In our proof‐of‐concept study, the segmentation variation was not evaluated; however, further study would be needed to investigate the impact of segmentation uncertainty in real clinical settings. Additionally, the placement of the wire may also introduce certain uncertainty and the degree of uncertainty may depend on the gradient level of the body surface.

In the algorithm, the location of the compensator tray and compensator thickness were obtained from the RT Plan DICOM file, thus the uncertainties of the snout positioning and the thickness variation of the compensator are not included in the air gap measurement. However, typically for passive scattering proton machines, the snout positioning is checked rigorously during machine QA and the compensator QA assures that the manufactured compensator thickness matches well with the plan.

For treatment sites, such as liver and lung, where the breathing motion could change the body surface, the reconstructed body surface using kV images will only represent one phase of the breathing circle, and it can differ from the average body surface. The difference will partially depend on the breathing magnitude of the surface.

The developed computer program uses the point on the centerline of the wire to compute the air gap values, which does not take the wire thickness into account, thus reducing the air gap value by the amount of the wire radius. For example, for the plan with the air gap value of 85 mm, while we originally arrived at a smaller air gap value of 82.62 mm using the outermost point on the wire, after taking into account the wire thickness (2.7 mm), the calculated air gap of 83.97 mm was derived as the distance from the skin surface to the end of the compensator.

In our calculation, we treat the end of the compensator tray or the snout as if it were a flat plane. While there could be other parts of the patient's body that are close to this plane, we only focus on the closest distance from the specific region of the skin that has the wire placed on it, at the angle used for treatment of the tumor.

On the other hand, with the implementation of surface imaging techniques in radiation oncology, including AlignRT^10^ (Vision RT, London, UK) and Catalyst^11^ (C‐RAD AB, Sweden), it is possible to capture the surface image with optical camera systems and monitor the air gap in real time. However, these optical systems may not be available on majority proton facilities and additional development and studies will be conducted in the next study.

## 5 CONCLUSIONS

An orthogonal image‐based method has been developed to automatically and objectively derive the beam air gap values in proton treatment, without adding additional imaging time and doses. Knowing the desired air gap during treatment allows us to confirm that the target depth will not exhibit significant change and will be properly covered by the proton dose and that the normal tissues along the beam path will not be unexpectedly compromised. The back‐projection algorithm and method described above is experimentally proven to be accurate. It is simpler, more efficient, and more easily accessible and reproducible than most other surface localization techniques currently available to proton centers equipped with only 2D imaging systems. Although a three‐dimensional positioning imaging device may be the ultimate approach to automatically compute air gaps and therefore the target depth, this developed method can be useful and beneficial for both the patient and the radiation oncology team for the proton systems which still rely on two‐dimensional imaging systems for patient treatment positioning.

## CONFLICT OF INTEREST

The authors have no relevant conflict of interest to disclose.
